# Supplementary material for: CDK1 Promotes Epithelial–Mesenchymal Transition and Migration of Head and Neck Squamous Carcinoma Cells by Repressing ∆Np63α-Mediated Transcriptional Regulation
Source: Int J Mol Sci. 2022 Jul 2;23(13):7385. doi: 10.3390/ijms23137385 (PMC9266818; doi:10.3390/ijms23137385)
Supplement: Supplementary file 1 [file ijms-23-07385-s001.zip › ijms-1790217-supplementary.pdf]

CDK1 promotes epithelial-mesenchymal transition and migration of head and neck squamous carcinoma cells via repressing △Np63α-mediated transcriptional regulation

Huimin Chen [1,#], Ke Hu [1,#], Ying Xie [1], Yucheng Qi [1], Wen-juan Li [2], Yao-hui He [2], Shijie Fan [1], Wen Liu [2], Chenghua Li [1,*]

1. Center of Growth, Metabolism and Aging, Key Laboratory of Biological Resources and Ecological Environment of Ministry of Education, College of Life Sciences, Sichuan University, Chengdu 610065, China
2. State Key Laboratory of Cellular Stress Biology, School of Pharmaceutical Sciences, Xiamen University, Xiamen, Fujian 361102, China

# These authors contribute equally

* Correspondence: lichenghua@scu.edu.cn

Legends for supplementary data

Supplementary Table S1. The list of identified △Np63α-interacting kinases.

HEK293T cells were transfected with Flag-tagged ΔNp63α. Cells were lysed using Cell lysis buffer for Western and IP (Beyotime) with Protease and phosphatase inhibitor cocktail for general use (Beyotime) at ice for 10 min. Lysates were cleared by centrifugation at 13,000 rpm for 15 min. Protein concentration was determined using the Bradford protein assay reagent (Coolaber). Then, 2 mg of total proteins from cell lysates were incubated with anti-Flag or normal mouse IgG as control at 4 °C for 8 h, and the immune complexes were precipitated with protein A+G-agarose at 4 °C for 2 h. The immunoprecipitates were washed with lysis buffer, separated by SDS-PAGE, and subjected to mass spectrometry (MS) as described previously [1]. The identified kinases were listed. MW, molecular weight. Calc. pI, calculated isoelectric point.

Supplementary Figure S1. The depiction of identified phosphorylated residues in △Np63α protein. The phosphorylated residues were depicted in △Np63α, according to the abovementioned mass spectrometry. TAD, transactivation domain; DBD, DNA binding domain; OD, oligomerization domain; SAM, sterile alpha motif; TID, trans-inhibition domain.

Supplementary Figure S2. The effects of mutations at the phosphor-sites on transactivity of △Np63α. The phospho-sites in DNA-binding domain (DBD) and trans-inhibition domain (TID) of △Np63α were substituted with alanine (A) to prevent the potential phosphorylation. The mutant and wild-type △Np63α constructs were subjected to luciferase reporter assay to assess the effects of these residues on △Np63α-mediated transcription. HEK293T cells were transfected with a mixture of

K14-Luc and TK-Renilla, plus △Np63α or its mutant plasmid as indicated. Firefly and Renilla luciferase activities were measured. The K14-Luc expression levels were normalized to Renilla activity and presented as means ± SD (n = 3). Two-tailed *t*-test was used for comparison between two groups; *, $p < 0.05$; **, $p < 0.01$.

Supplementary Figure S3. The fragment of △Np63α containing T123 is a conservative motif fitting the consensus sequence for CDK1 modification. The fragment containing T123 fits the consensus sequence for CDK1 according to the prediction using Eukaryotic Linear Motifs (ELM, website: http://elm.eu.org).

Supplementary Figure S4. △Np63α directly binds to promoters of BPAG1 and N-cadherin. **(A)**, The binding site of △Np63α (S1) is contained in the BPAG1 promoter as reported previously [2]. TSS, transcription start site. **(B)**, There are 3 putative binding sites of △Np63α (S1, S2 and S3) in the N-Cadherin promoter as predicted with JASPAR online software (https://jaspar.genereg.net/analysis). TSS, transcription start site. **(C)**, Chromatin immunoprecipitation (ChIP) assays were performed in UM1 cells stably overexpressing Flag-ΔNp63α or Flag-ΔNp63α plus CDK1, with anti-Flag. DNA samples precipitated with either anti-Flag or mock IgG, as well as equivalent input, were subjected to polymerase chain amplification (PCR) to detect fragments of BPAG1 and N-cadherin promoters.

Supplementary Table S1.

Name	Description	MW (KDa)	Calc. pI	Score
AURKB	Aurora kinase B	39.3	9.29	2.45
CDK1	Cyclin-dependent kinase 1	34.1	8.4	35.6
CDK4	Cyclin-dependent kinase 4	33.7	7.01	14.04
CDK16	Cyclin-dependent kinase 16	55.7	7.62	13.26
CHK2	Checkpoint kinase 2	60.9	5.91	162.33
CK1α1	Casein kinase I isoform alpha	38.9	9.57	24.24
CK1δ	Casein kinase I isoform delta	47.3	9.74	40.09
CK1ε	Casein kinase I isoform epsilon	47.3	9.66	42.7
CK2α1	Casein kinase II subunit alpha	45.1	7.74	21.25
CK2α2	Casein kinase II subunit alpha'	41.2	8.56	16.59
CK2β	Casein kinase II subunit beta	24.9	5.55	22.49
JAK1	Janus tyrosine kinase 1	133.2	7.55	17.3
MAP3K7	Mitogen-activated protein kinase kinase kinase 7	67.2	7.11	12.37
MAPK1	Mitogen-activated protein kinase 1	41.4	6.98	70.53
RIOK1	RIO serine/threonine kinase 1	65.5	6.19	57.61
SRPK2	SRSF protein kinase 2	77.5	4.97	5.52
STK38	Serine/threonine-protein kinase 38	54.2	7.15	205.52

Supplementary Figure S1

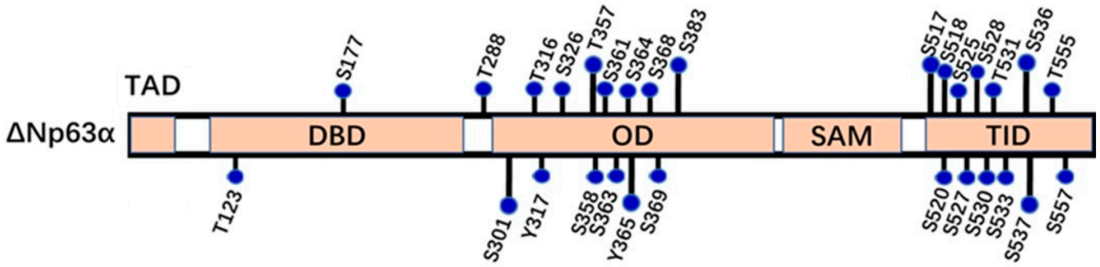

Supplementary Figure S2

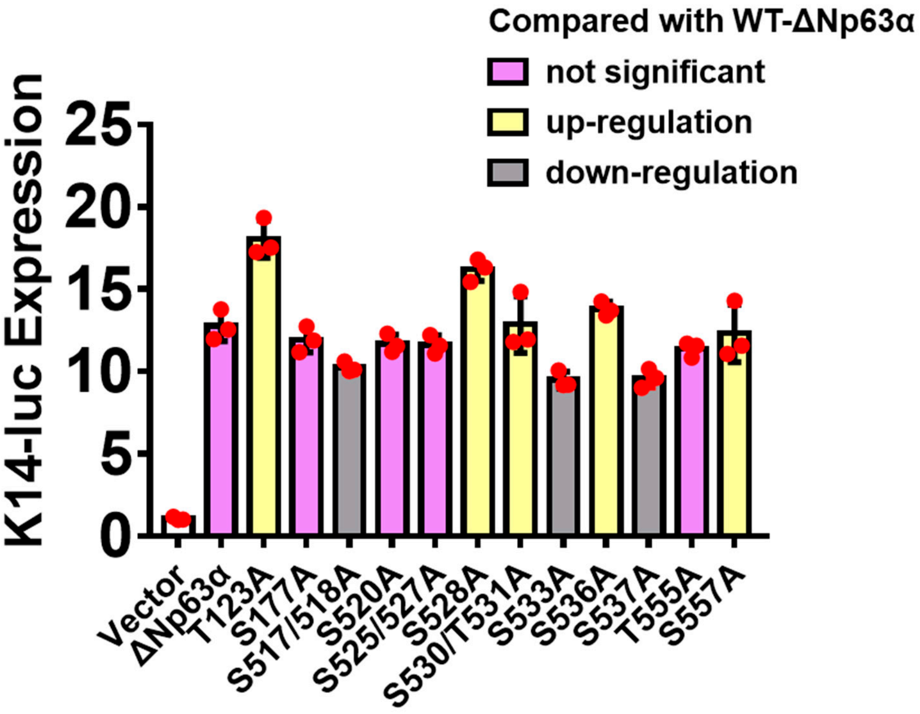

Supplementary Figure S3

Consensus sequence for CDK1 modification: SP or TP

Homo ΔNp63α	**118 QIKVMTPPPQGAVI 131**
Mus ΔNp63α	**118 QIKVMTPPPQGAVI 131**
Rat ΔNp63α	**118 QIKVMTPPPQGAVI 131**
Xenopus ΔNp63α	**118 QIKVMTPPPQGAVI 131**

Supplementary Figure S4

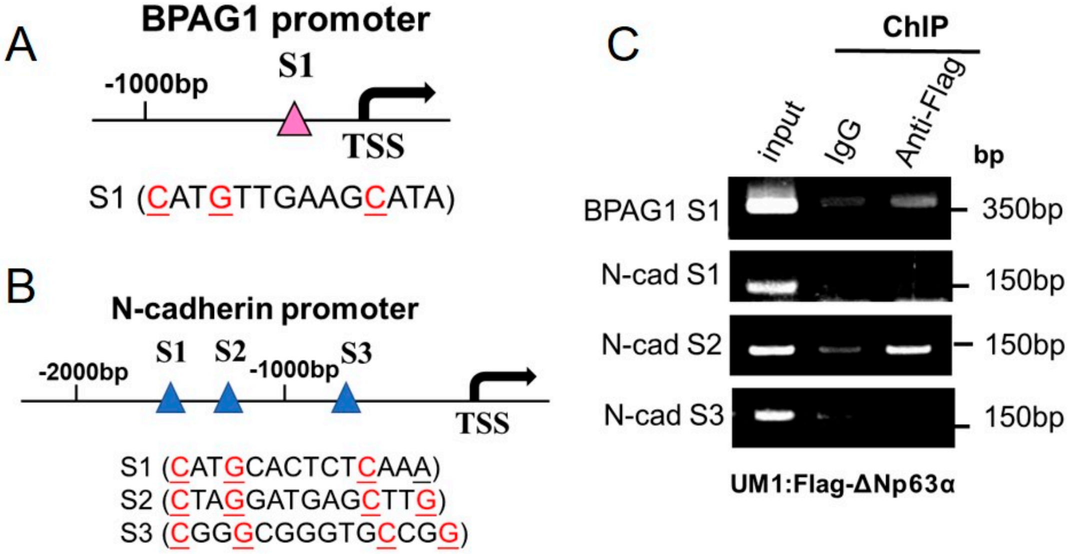

References:

1. Peng, B. L.; Li, W. J.; Ding, J. C.; He, Y. H.; Ran, T.; Xie, B. L.; Wang, Z. R.; Shen, H. F.; Xiao, R. Q.; Gao, W. W.; Ye, T. Y.; Gao, X.; Liu, W., A hypermethylation strategy utilized by enhancer-bound CARM1 to promote estrogen receptor alpha-dependent transcriptional activation and breast carcinogenesis. *Theranostics* **2020,** *10* (8), 3451-3473.
2. Osada, M.; Nagakawa, Y.; Park, H. L.; Yamashita, K.; Wu, G.; Kim, M. S.; Fomenkov, A.; Trink, B.; Sidransky, D., p63-specific activation of the BPAG-1e promoter. *J Invest Dermatol* **2005,** *125* (1), 52-60.
